# Online and personalised control of the Depth of hypnosis during induction using fractional order PID

**DOI:** 10.1016/j.jare.2025.03.053

**Published:** 2025-03-29

**Authors:** Marcian Mihai, Isabela Birs, Erwin Hegedus, Amani Ynineb, Dana Copot, Robain De Keyser, Clara M. Ionescu, Samir Ladaci, Cristina I. Muresan, Martine Neckebroek

**Affiliations:** a Technical University of Cluj-Napoca, Department of Automation, Cluj-Napoca, Romania; b Ghent University, Department of Electromechanics, Systems and Metal Engineering, Research Group on Dynamical Systems and Control, Tech Lane Science Park 125, Gent 9052, Belgium; c Ecole Nationale Polytechnique, Department of Electronics, 10 Rue des Frères Oudek, El Harrach 16200 Algiers, Algeria; d Ghent University Hospital, Department of Anesthesia, C. Heymanslaan 10, 9000 Ghent, Belgium

**Keywords:** Drug dosing, Anaesthesia, Closed loop control of anaesthesia, Fractional-order models for hypnosis, Delay time estimation, Online fractional order control, Personalised anaesthesia

## Abstract

**Introduction:**

The integration of information technology and control engineering has significantly increased in clinical practice research, especially in the management of drug dosing for general anaesthesia.

**Objective:**

The main focus of the research is to achieve a personalised control of the Depth of Hypnosis based on a novel approach. The primary objective is to regulate the Bispectral Index by administering Propofol during the hypnotic phase of anaesthesia.

**Methods:**

A fractional order time delay model is proposed to replace the conventional PK-PD model. This model is estimated online during the induction phase. A fractional order Proportional-Integral-Derivative (PID) controller is then individually tuned for each patient.

**Results:**

The closed loop simulation results demonstrate that the proposed controllers effectively minimise undershoot and achieve a short time to reach the desired time-to-target for the majority of the patients. The analysis indicates that the proposed controller is capable of maintaining the BIS signal within a safe range. Comparative analyses with similar research are provided to effectively contextualise this study within the existing literature. Disturbance rejection tests during the maintenance phase are performed to demonstrate the effectiveness of personalised control.

**Conclusion:**

The online adaptation that this research brings, compared to the previous attempts, allows for significantly improved results for the hypnosis phase.

## Highlights

• Development of fractional-order models time delay models for hypnosis.
• Online estimation of time delay and personalised design of fractional order PID control for drug dosing.
• Automatic control of hypnosis during idnuction phase.
• Closed loop control of anaesthesia.

## Graphical abstract

**Figure:**
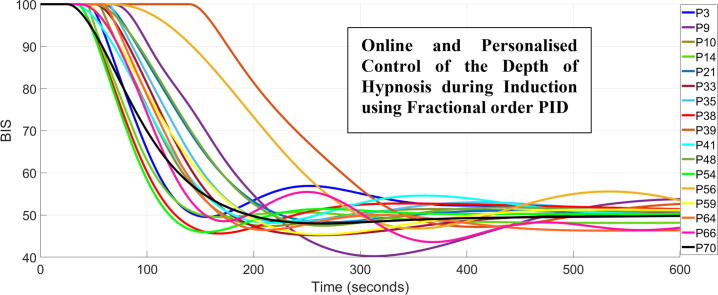

## Introduction

Target controlled infusion (TCI) has become increasingly popular in recent years due to their extensive use in operating rooms during anaesthesia procedures. In TCI techniques, the delivery of the anaesthetic drugs is performed using an infusion pump. The infusion rate is calculated according to some specific patient parameters, such as age, weight, height, gender, to name just a few, with the end purpose of achieving either a certain plasma concentration of the drug or a specific site effect. The calculation of infusion rates is based on a three-compartmental pharmacokinetic (PK) model, combined with a pharmacodynamic (PD) model [1]. Even though TCI systems have been used for more than two decades, there is still an ongoing debate regarding the accuracy of the PK models, with notable differences between the various approaches [2]. PK models variants use different formulas to account for patient characteristics, such as body mass integration, the use of gender and age, etc. Usually, they are limited to a certain group of patients and some PK models work well for some patients and poorly for others [2]. An additional issue refers to the great patient variability, which is yet another problem when determining an appropriate drug dosage. Thus, due to the unpredictable and distinct attributes of the human body [3], [4], customised patient models are necessary. The development of an online and personalised model of the patient would be therefore beneficial for a more accurate computation of the infusion rates. This is one of the main tasks and contributions of the present work.

With the emergence of new and modern patient monitors and infusion pumps, closed loop TCI and Total IntraVenous Anaesthesia (TIVA) have become more accurate, safe and easy [5]. One of the key patient vital signs that are monitored and controlled during TIVA and closed loop TCI is the Depth of Hypnosis, evaluated using the Bispectral Index (BIS) [6], [7]. The administered drug is Propofol. This paper aims to optimise the hypnosis state for individual patients, with a particular focus on the significance of administering the appropriate dosage of Propofol to achieve the optimal hypnosis state. To achieve this end, a personalised controller is developed based on an online identified patient model.

Several control algorithms have been previously developed for managing the BIS and optimizing Propofol infusion rates. A great majority focuses on Proportional-Integral Derivative (PID) controllers. In [8], a PID is tuned such that the integral of absolute error (IAE) is minimized. The designed PID controller is tested on 12 different patients. A multiobjective optimization routine is used in [9] to design PID controllers for a combined anaesthesia and hemodynamic system, including BIS. PID controllers for regulating the Depth of Hypnosis using a co-administration procedure of two drugs, including Propofol, have been also developed as viable alternatives to the standard approach [10], [11], [12], [13], [14]. Although an online identification of the dose–effect response is performed, the simulation results in [14] show the limitations of the co-administration approach, claiming that this is suitable only during the induction phase of general anaesthesia. Extensions of the PID algorithm have been also developed, such as a PIDA (PID-Acceleration) controller [15] and the results have been tested both during the induction and maintenance phases of anaesthesia. Model based predictive control (MPC) algorithms have been also proposed as suitable replacements for PID control. Several papers discuss the potential of MPC in regulating BIS, such as [16], [17], [18], [19], [20]. Others have focused on fuzzy logic control [21], [22]. Recent papers have discussed the potential of using fractional calculus in designing generalized fractional order PID controllers for regulating the Depth of Hypnosis [23], [24], [25]. Different other control algorithms used in automated anaesthesia can be found in a very recent review [26].

All these control algorithms have one common thing: the need of a mathematical model for the patient. While most research consider population-based models, some have moved in the direction of personalised control, such as [12] or [27]. In [12], an optimization routine is used to tune a PID controller based on the covariates of the PK model for propofol. Based on this approach an optimal set of tuning parameters is obtained for each combination of patient demographics. This ultimately leads to individualized controller parameters based on patient demographics. The closed loop simulation results have shown that the individualized controller provides better robustness to patient variability, but also exhibits a decrease in the bandwidth, which results in poorer disturbance rejection capabilities. The PK model employed here links the Propofol infusion rate to the effect-site concentration. However, a PD model is additionally required to fully described the effect of Propofol on BIS signal, as it links the effect-site concentration to the effective BIS. Quite frequently, the PD model is mathematically described using a Hill function. Unlike the PK model, the parameters of the PD model do not depend on the patient demographics and are usually subjected to large variability [12]. The PID controller in [12] is designed to handle covariates in the PK model solely and thus represents a limitation towards achieving personalised control. In [27], the authors use the Eleveld PK-PD model, which is considered to be more accurate in describing the dynamics between the Propofol infusion rate and the Bispectral Index. For each patient, the demographics are used to create a PK-PD model. This is later used to tune an individualised PID controller using an optimization routine that minimizes the IAE. The closed loop simulation results show that the individualised control strategy outperforms the population-based controller. However, limitations to the individualised controller design refer exactly to the PK-PD model, which has not been developed for control purposes.

In this paper, a similar approach to [12] or [27] is considered. The patient BIS signal is recorded during the induction phase. The collected data is then used to estimate a fractional order plus dead time model, which replaces both the PK and PD models from classical approach. In contrast to [12] and [27], which use an offline PK-PD model for the patient, the fractional order model is estimated online during the induction phase. As such, designing a controller using the fractional order model is indeed individualised and specific to each patient. Fractional order models have been used before in biomedical applications such as the model of a tumour-immune surveillance mechanism [28], virus transmission [29], [30], [31], electrical properties of nerve cell membranes and the propagation of electrical signals [32], nociception and analgesia [33], [34], viscoelastic phenomena occurring in musculoskeletal tissues [35] or lung tissues [36], [37], dynamics of smoking [38] to name just a few. Drug diffusion has also been extensively studied and several fractional order models have been proposed as being more suitable compared to classical integer order approaches [39], [40]. Fractional order pharmacokinetic models have also emerged and successfully used to fit clinical data [41], [42]. The drug dynamics in time and within the human body follows the anomalous diffusion mechanism. As such, fractional calculus has been regarded as more suitable to describe this dynamic. Additionally, the use of fractional calculus leads to new and potentially useful functional relationships for modelling complex biological systems in a direct and rigorous manner [32]. This justifies the development of a fractional order transfer function with time delay to model the patient’s response from Propofol to BIS, attempted in this paper.

Once an online fractional order model for the patient has been obtained, an online and personalised controller needs to be designed. Previous research has demonstrated the advantages of fractional order PID (FOPID) controllers over integer order PIDs [43], in terms of increased robustness and flexibility, better handling of disturbances, improved control for time delay systems, etc [44]. The proposed FOPID controller has a novel structure compared to previous research [23], [24], [25]. The parameters of the FOPID are tuned using the newly developed fractional order models. The controller underwent testing on models of 20 patients. They represent a database of patients participating in a clinical trial. This academic clinical investigation received approval from the Ethics Committee of Ghent University Hospital and the Federal Agency for Medicines and Health Products of Belgium (FAGG) under the codes EC/BC-08020 and FAGG/80 M0840. The study is registered under the identifiers EudraCT: CIV-BE-20-07-0342442020 and clinicaltrials.gov: NCT04986163, with Martine Neckebroek serving as the principal investigator. Further details regarding the clinical trial can be found in [33].

The importance of online modelling and control is emphasised in references [46], [47]. Precise and timely estimation of system parameters is crucial for optimising performance and ensuring stability in biomedical engineering and drug dosing applications. The paper highlights the successful online identification of second-order system coefficients by employing metaheuristic algorithms, which are renowned for their strong optimisation capabilities. The ability to estimate parameters in real-time allows for flexible adjustments in control strategies, enabling systems to adapt to changing conditions and disturbances. Automated systems are improved in terms of efficiency and reliability, leading to progress in all control engineering areas, and specifically in biomedical engineering and drug dosing solutions, where precision and adaptability are of utmost importance.

The most important contributions and novel elements of this research consist in:

• The development of fractional order time delay transfer functions to model the BIS-Propofol dynamics and replace the traditional PK-PD models, which have not been developed for control purposes
• The online estimation of the fractional order model parameters using clinical data during the induction phase of anaesthesia
• The design of a novel personalised FOPID controller, with a novel structure compared to previous research
• The implementation and analysis of closed loop results during the induction and maintenance phases

The paper is structured as follows: Next section present the fractional order modelling approach, and the delay time estimation part, which delivers the complete model of the patient. Further, the design of the tailored controllers, according to each patient model are presented. The findings and their relevance together with the limitations are displayed in the results and discussion section. The last section highlights the conclusion of this work.

## Fractional order modelling

A fractional order (FO) model has been developed to mathematically describe the effect of Propofol on the Depth of Hypnosis (DoH) evaluated through the measured BIS signal. Prior studies have demonstrated that the BIS signal exhibits a gradual decline from a fully alert state (represented by a value of 100) to a state of hypnosis, indicated by a BIS signal ranging from 40 to 60. The optimal dosage of medications is frequently predicted using compartmental models, which are the foundation of TCI management systems in clinical practice [48], [49]. The compartmental modelling approach posits that the drug is administered intravenously to the blood compartment and subsequently diffuses into the muscle and fat compartments before arriving at a theoretical effect site compartment. The set of differential equations which describes this compartmental PK model is found in (1).

(1) $$\begin{array}{c} {\dot{x}}_{1}\left(t\right)=-\left(k_{10}+k_{12}+k_{13}\right)x_{1}\left(t\right)+k_{21}x_{2}\left(t\right)+k_{31}x_{3}\left(t\right)\\ {\dot{x}}_{2}\left(t\right)=k_{12}x_{1}\left(t\right)-k_{21}x_{2}\left(t\right)\\ {\dot{x}}_{3}\left(t\right)=k_{13}x_{1}\left(t\right)-k_{31}x_{3}\left(t\right), \end{array}$$

where *x_1_*(*t*) denotes the drug concentration in the blood compartment, *x_2_*(*t*) denotes the drug concentration in the muscle compartment, and *x_3_*(*t*) denotes the drug concentration in the fat compartment. Parameters *k_ij_* represent the drug transfer rates from the *i*^th^ to the *j*^th^ compartment. Applying the Laplace transform to (1) leads to simple first order transfer functions. The blood compartment, ${\dot{x}}_{1}\left(t\right)$, is the central one. The muscle compartment, ${\dot{x}}_{2}\left(t\right)$, exhibits considerably faster dynamics than the central compartment. As such, from a control engineering point of view, the muscle compartment exhibits a significantly smaller time constant compared to the one that characterises the central compartment and can thus be neglected. The fat compartment, ${\dot{x}}_{3}\left(t\right)$, is known as the slowest one, leading to a lethargic effect close to the steady-state value and with a minimal effect upon the overall dynamics, which is primarily governed by the dynamics of blood compartment. The integer order model in (1) can then be reduced to a first-order transfer function characterised by the time constant *T_1_*, specific to the blood compartment. The effect site compartment is described by:

(2) $${\dot{x}}_{e}\left(t\right)={-k}_{0e}x_{e}\left(t\right)-k_{1e}x_{1}\left(t\right)$$

where $k_{e0}$ is the drug metabolic rate and $k_{1e}$ is the drug transfer rate from the blood compartment to the effect site compartment. Applying the Laplace transform on (2) and rewriting it as a transfer function leads to:

(3) $$\frac{x_{e}\left(t\right)}{x_{1}\left(t\right)}=-\frac{\frac{k_{1e}}{k_{0e}}}{\frac{1}{k_{0e}}s+1}$$

The last compartment can be modelled into a first order transfer function having the time constant $T_{2}=\frac{1}{k_{0e}}$. The pharmacodynamic component of the model is mathematically expressed through a non-linear Hill curve, which is ultimately simplified to a gain in steady state. This models the diffusion of the drug from the effect site to its final effect. Recent findings suggested that the DoH sensors introduce a variable time delay, thus leading to a time delay mathematical model [27]. Considering all the aforementioned factors, the final simplified integer order transfer function for DoH is represented as a damped second-order system of the following form:

(4) $$H_{\mathrm{DoH}}\left(s\right)=\frac{k}{\left(T_{1}s+1\right)\left(T_{2}s+1\right)}e^{-\tau_{m}s}$$

where $\tau_{m}$ represents the time delay. The second order model in (4) is further altered to a fractional order model, to better approximate the anomalous diffusion of the drug in the human body [32]. Consequently, the proposed mathematical model is indicated as follows:

(5) $$H_{\mathrm{DOH}}\left(s\right)=\frac{\mathrm{BIS}(s)}{\mathrm{PROP}(s)}=\frac{k}{a_{2}s^{\alpha_{2}}+a_{1}s^{\alpha_{1}}+1}e^{-\tau_{m}s}$$

where BIS(s) and PROP(s) are the Laplace transforms of the BIS signal and Propofol progression rate, a_2_ and a_1_ are coefficients and $\alpha_{1}$ and $\alpha_{2}$ are the fractional orders. The use of a reduced parameter model will facilitate the design of the control algorithm by decreasing computational effort.

The implementation of the fractional order model in (5) is achieved using the Grunwald-Letnikov approximation of the fractional derivative:

(6) $$D^{\alpha }f\left(x\right)=h^{-\alpha }\sum_{n=0}^{\infty }-1^{n}\left(\begin{array}{c} \alpha \\ n \end{array}\right)f\left(x-n∙h\right)$$

where *D^α^* represents the fractional order derivative operator, *α* is the fractional order and *h* represents the step size or the spacing between the points in the function.

An optimization routine was implemented to estimate the parameters in (5). The conventional Levenberg-Marquardt algorithm (LMA) effectively estimates the parameters of the delay free model in (5) by minimising the sum of squared errors between the measured BIS output and the calculated BIS signal. The time delay is estimated using a simple procedure, that is described in the following section of the paper. This study developed an adaptation of the LMA, similar to [50], [51]. Models which derive from solving a system of error-equations are linear in coefficients. The proposed system in (5) is characterized by the parameters vector:

(7) $$\theta ={\left[ka_{2}a_{1}\alpha_{2}\alpha_{1}\right]}^{T}$$

The dead time, $\tau_{m}$, is estimated independently of the other parameters. The algorithm possesses a generic structure and is not limited to the specific instance of FO transfer functions as outlined in (5). The estimated output expressed in regression form is:

(8) $$\hat{\mathrm{BIS}}\left(t\right)=\phi \left(t\right)∙\theta$$

where the parameter vector was previously defined and the regression vector *ϕ* is given by:

(9) $$\phi \left(t\right)=\left[\begin{array}{ccc} D^{\alpha_{0}}PROP\left(t\right) & \cdots & D^{\alpha_{n}}PROP\left(t\right)\\ \vdots & \ddots & \vdots \\ {-D}^{\beta_{0}}BIS\left(t\right) & \cdots & {-D}^{\beta_{n}}BIS\left(t\right) \end{array}\right]$$

The partial derivatives in (9) are computed according to (6). Let *E* be the error vector. The cost function, *F*, to be minimised in order to obtain the estimated parameters vector, $\hat{\theta }$, is defined as:

(10) $$F\left(\hat{\theta }\right)=E^{T}E$$

Let *J* be Jacobian matrix of the system. LMA introduces a novel damping factor, *μ*. An update is made at each step-size, *δ*, in the pursuit of the minimum:

(11) $$\left(J^{T}J+\mu I\right)\delta =J^{T}\left|BIS-\hat{\mathrm{BIS}}\left(\hat{\theta }\right)\right|$$

where *I* denotes the identity matrix. This is the refinement brought to the classical least squared method.

To assess the accuracy of the models, we start by calculating the average value $\overset{¯}{\mathrm{BIS}}$ of the measured BIS signal using the following formula:

(12) $$\overset{¯}{\mathrm{BIS}}=\frac{1}{n}\sum_{k=1}^{n}{\mathrm{BIS}}_{k}$$

where *n* is the maximum number of samples considered in the estimation of the model parameters and BIS_k_ denotes the measured BIS output at each sample *k*. The fit of the FO model is computed using the “R^2^” method [52]. The goodness of fit is calculated as in (13):

(13) $$\begin{array}{c} {\mathrm{SS}}_{\mathrm{res}}=\sum_{k=1}^{n}{\left({\mathrm{BIS}}_{k}-{\hat{\mathrm{BIS}}}_{k}\right)}^{2}\\ {\mathrm{SS}}_{\mathrm{tot}}=\sum_{k=1}^{n}{\left({\mathrm{BIS}}_{k}-\overset{¯}{\mathrm{BIS}}\right)}^{2}\\ fit=100∙\left(1-\frac{\mathrm{SSR}}{\mathrm{SST}}\right)\left(\%\right) \end{array}$$

where SSR means the residual sum of squares, and SST represents the total sum of squares.

The study employs a database consisting of 20 patients with recorded input/output signals (Propofol progression rate, respectively Bispectral Index). For each patient, clinical input and output data is used to estimate a fractional order model of the form indicated in (5) using the proposed methodology based on LMA. The estimated parameters of the FO model for each of the 20 patients are provided in Table 1. The resulting fit exceeds 80 % in almost all cases, with the exception of patient P70. In each of the examined instances, the patient was administered a bolus of Propofol at a dosage of approximately 140 mg/kg/h for a short duration, resulting in the initiation of hypnosis (induction phase). This was followed by a reduced drug dosage to maintain the BIS signal within the 40–60 range.

**Table 1:** Estimated parameters of the fractional order models for depth of hypnosis for 20 patients.

P. No.	k	a_2_	a_1_	α_2_	α_1_	$\tau_{m}$	Fit%
3	5.04	2500	179.43	1.81	0.76	45	82.2
9	4.70	41,700	950.00	2.86	1.19	65	86.4
10	1.89	29,700	342.12	1.86	0.94	60	85.0
14	2.55	0.89	396.58	1.93	0.89	45	85.8
21	5.71	3480	375.18	1.96	0.96	60	82.8
25	0.56	2550	100.58	1.69	1.67	85	82.7
33	4.62	43,900	1614.8	2.15	1.15	50	85.5
35	4.29	0.37	1233.1	2.13	1.08	65	82.7
36	8.22	34,900	3988.9	2.24	1.06	160	82.1
38	1.87	4500	82.18	1.72	0.86	35	83.9
39	2.55	69,300	294.23	2.05	0.98	140	86.2
41	2.31	60,300	418.18	2.14	0.95	30	86.6
48	2.19	44,700	769.42	2.21	1.13	50	85.3
54	1.66	8830	112.88	1.87	0.91	35	86.2
56	0.77	16,700	216.09	2.36	1.20	65	87.1
59	5.24	3290	1440.4	2.52	1.19	45	86.0
63	1.01	39,800	271.30	3.58	1.03	90	86.3
64	6.93	22.90	340.70	3.25	0.84	60	86.2
66	0.46	3880	51.83	2.31	0.99	35	87.9
70	6.66	91,700	149.7	2.05	1.04	25	79.7

**Fig. 1:**
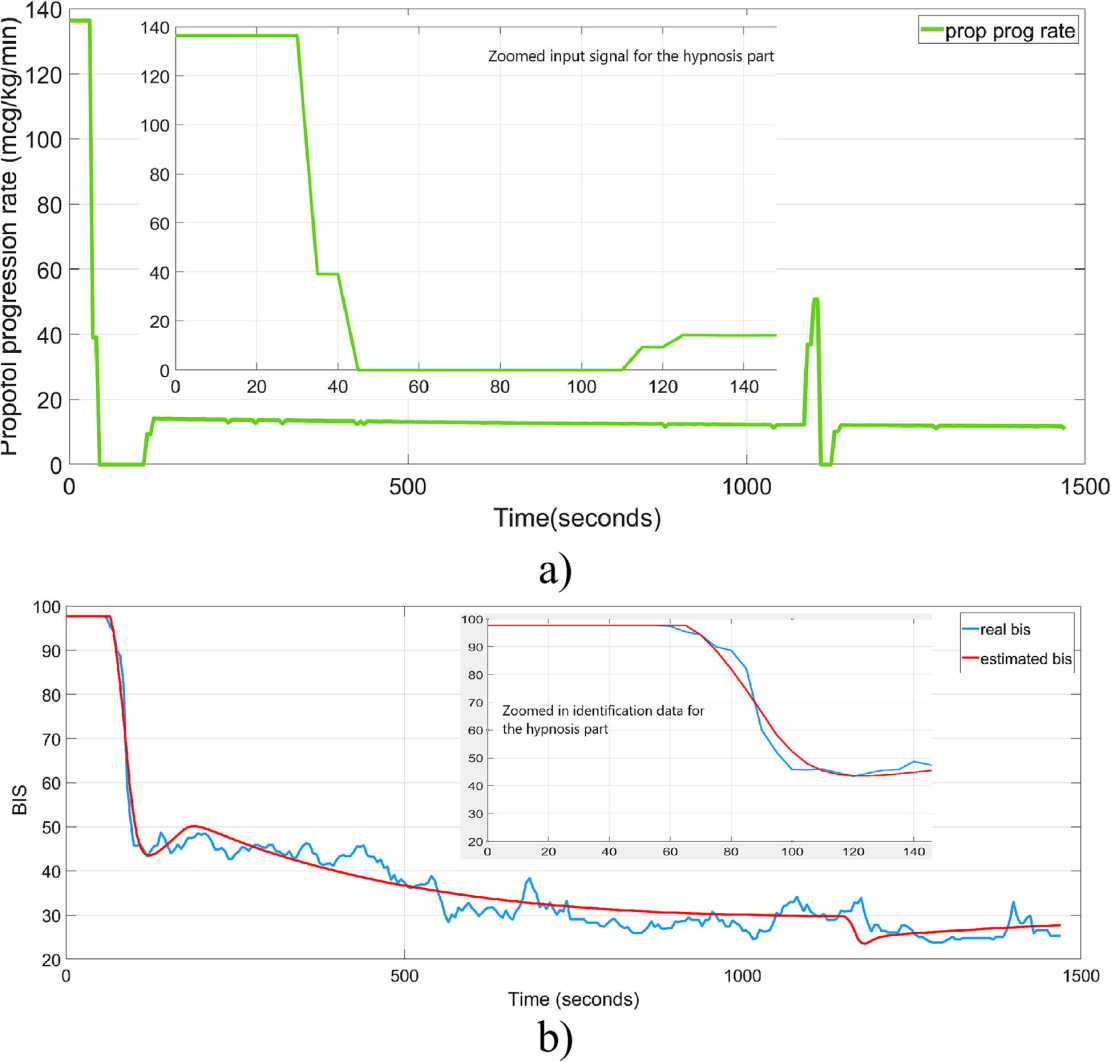
a) Propofol drug rate administered to patient 21 (green) b) Measured BIS signal (blue) and estimated BIS signal (red).

An example of clinical data compared to the simulated FO model is indicated in Fig. 1, for patient 21 in the database. The drug given to patient 21 is specified in Fig. 1a), while Fig. 1b) shows the corresponding progression of the BIS signal for the same patient. The parameters of the FO model in (5), corresponding to patient 21, are indicated in Table 1. The parameter estimation accuracy for the FO model in patient 21 is 82.8 %. The calculated value of the BIS signal, obtained from the model described in equation (5), is also shown in Fig. 1b. It is important to mention that the measured BIS signal (in blue) has a significant amount of noise, but the trend shows patterns that are accurately represented by the estimated FO model of the form given in (5).

Similar results have been obtained for the remaining patients in the database, using the available Propofol and BIS clinical data. The parameters of the FO models corresponding to each patient have been estimated online using the methodology described in this section, based on the adapted LMA algorithm. In each case, the resulting fit is indicated in Table 1.

A statistical analysis considering the average value of the parameters and the deviation is performed next. Based on Table 1, the average value for the gain is *k_mean_* = 3.46, while the average values for the denominator coefficients are *a_2mean_* = 25087.71 and *a_1mean_* = 666.38. The mean values for the fractional order are $\alpha_{1mean}=1.05$ and $\alpha_{2mean}=2.23$. The average time delay is estimated to be $\tau_{m}$ = 62.2 s. In terms of deviations from the average values, coefficient variations are ranging from a decrease of 99 % to an increase of 276 % for *a_2_*, and an increase of 598 % for *a_1_*, while the gain ranges from −87 % to + 237 %. The fractional order deviates from its nominal values by approximately −25 % to 60 %, while the time delay varies within a range of −60 %–160 %. This suggest a great patient variability that needs to be accounted for using a personalised controller that is tuned online, based on the online estimated patient model.

A population based robust fractional order controller has been previously designed in [24]. However, due to large patient variability, indicated in Table 1, this controller has proved to be inefficient for a majority of the patients in the database. In fact, satisfactory closed loop results were obtained for merely 8 out of 22 individuals (36.36 %) [24]. An online tuned controller has been deemed necessary and has been effectively implemented in this paper. The term “online controller” refers to the design of a personalised controller for each patient, with the controller parameters computed online during the induction phase.

## Time delay estimation

The accurate estimation of the time delay is particularly important in the design of the FOPID controller, as it directly and significantly affects both the settling time and the stability of the closed-loop system. Two different algorithms for time delay estimation have been developed and compared in this section. Both algorithms are simple and efficient. To compare the efficiency of the algorithms, a performance metric is used, i.e. the Root Mean Square Error (RMSE) [52]. The mathematical form of the RMSE is:

(14) $$\mathrm{RMSE}=\;\sqrt{\frac{\sum_{i=1}^{n}{\left({\tau_{m}}_{i}-{\hat{\tau_{m}}}_{i}\right)}^{2}}{n}}$$

where *n* denotes the number of samples in the database, $\tau_{m}$ and $\hat{\tau_{m}}$ represent the actual time delay, respectively the estimated time delay.

The core principle of the algorithms lies in the estimation of the exact moment when the patient begins to respond to the initial Propofol bolus administration, resulting in a significant decrease in the BIS signal. The algorithms can be summarised as follows: identify the genuine deviation in the BIS signal, distinguishing it from false oscillations or noise. Then, repeatedly revert a specified number of samples to the initial signal value until the signal returns to its original state. The halting point is indicated by the index, which is decreased by one sample, in the temporal vector where the estimation of the delay time is performed. The difference between the two algorithms is the following. The first one uses the first value from the BIS vector, while the second algorithm relies on the mean value of the first few samples.

**Fig. 2:**
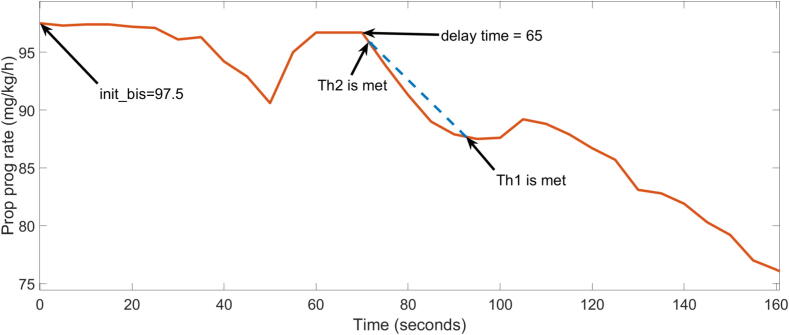
Delay time estimation in graphical representation.

The pseudocode of the first algorithms are indicated next. The time vector is discrete, with all samples evenly spaced at intervals of 5 s. The temporal instant associated with index ‘*I*’ is calculated by multiplying ‘*I*’ by 5 and subtracting 5. To determine the specific moment when the BIS signal decreases, an additional index ‘*j*’ is used to help analyse the data retrospectively. The algorithm incorporates two distinct thresholds, *Th1* and *Th2*, which are crucial tuning parameters. *Th1* refers to the minimum level of change in the BIS signal that is required to differentiate true responses to the Propofol stimulus from possible false disturbances caused by noise or oscillations. The optimal empirical value determined was set at 10 % relative to the initial BIS value. Simultaneously, *Th2* plays a crucial role in identifying the beginning of the BIS variation to the input signal, Propofol. The algorithm first determines the index at which the change in the signal exceeds the threshold value *Th1*. This does not correspond to the actual delay time. To precisely identify the point at which the initial response occurs, the algorithm performs a retrospective backtracking operation, using *Th2*, which indicates the beginning of the stationary (dead) zone. This facilitates the estimation of the beginning of the BIS variation. The vector *BIS()* contains the consecutive values of the BIS. The output of the algorithm is the estimation of the dead time, referred to as ‘delay’ in the pseudocode. A graphical representation of the algorithm is given in Fig. 2.

**Table:** 

Delay estimation algorithm 1
1: Take the initial value from the BIS vector. Denote it with: init_bis
2: Establish a threshold of 10 % from the initial value. Denote it: Th1
3: Establish a threshold of 1 % from the initial value. Denote it: Th2
4: **for** I = 3 to n **do**
5: Find I for which init_bis – BIS(i) > Th1
6: **for** j = 0 to (i-1) **do**
7: Find j for which init_bis – BIS(i-j) < Th2
Denote est_delay = t(i-j-1)
**break** the loop
8: delay = est_delay

**Table:** 

Delay estimation algorithm 2
1: Take the initial 5 samples from the BIS vector. Compute their mean value and denote it with: mean_bis = mean(BIS(1:5))
2: Establish a threshold of 5 % from the initial mean value. Denote it: Th1
3: Establish a threshold of 1 % from the initial mean value. Denote it: Th2
4: **for** I = 3 to n **do**
5: Find I for which abs(mean_bis – BIS(i)) > Th1
6: **for** j = 0 to (i-1) **do**
7: Find j for which abs(mean_bis – BIS(i-j)) < Th2
Denote est_delay = t(i-j-1)
**break** the loop
8: delay = est_delay

The second time delay estimation approach employs the mean value as its initial parameter and differs from the first approach in terms of the starting value and the thresholds used in the algorithm. The underlying logic remains consistent in both methods. The two versions exhibit clear differences. The initial iteration exhibits a precision level of 70 % in estimating the delay, with a margin of error limited to one sample. Moreover, this version attains a precision of 85 % while permitting a maximum deviation of three samples. On the other hand, the second version exhibits a 60 % accuracy rate with a single sample error tolerance yet achieves a significantly higher accuracy of 90 % when the error tolerance is increased to three samples. The RMSE performances are *RMSE_1_* = 18.3 for the first algorithm and *RMSE_2_* = 13.37, for the second one. The performance figures are derived from the data in Table 2, and they suggest that the 1st version is generally more precise. On the contrary, the RMSE metric suggest that on average, the 2nd version is closer to the real value. The faulty estimation of patient 25 increases substantially *RMSE_1_*.

**Table 2:** Comparison between actual and estimated delays.

P. No.	Actual Delay (seconds)	Estimated Delay – Version 1 (seconds)	Estimated Delay – Version 2(seconds)
3	45	40	40
9	65	70	65
10	60	60	60
14	45	50	50
21	60	55	55
25	85	135	70
33	50	65	60
35	65	30	55
36	160	205	205
38	35	40	40
39	140	145	145
41	30	45	45
48	50	65	65
54	35	40	35
56	65	65	65
59	45	50	35
63	90	95	85
64	60	60	60
66	35	30	15
70	25	30	30

Limitation to the time delay estimation algorithms exist. In some cases, such as for patients 25 and 35, the results suggest that the second version of the algorithms is more suitable, while for the others the first version of the algorithm seems to be more appropriate. The effectiveness of the algorithm is constrained by the size of the patient database and the amount of clinical data available for this study. To reach a definitive conclusion regarding the optimal version of the algorithm, a more extensive dataset of patients would be necessary. Therefore, although initial results support the first version of the algorithm in terms of accuracy, the potential suitability of the second version in specific situations cannot be disregarded without additional research. In what follows, the first algorithm is used for the online time delay estimation.

## The online FOPID controller

Previous results considering a population-based controller [24], led to poor closed loop simulation results for most of the patients in the database. As mentioned before, in only 8 cases out of the 22 patients, good performance was obtained. The efficiency of the population-based controller is thus only 36.36 %. To increase the efficiency of the control algorithm, the approach in this paper was developed consisting in an online estimation of a patient model as in (5) followed by the online design of a FOPID controller, personalised to each individual. In this approach, the tuning of the fractional order controller is no longer performed based on the model of a selected nominal patient, but based on each patient’s model, identified online during the induction phase.

The tuning algorithm is based on the minimisation of a cost function along with constraints. For tuning the fractional order controller, the modulus (*M*), phase (*P*) and derivative of the phase ($\frac{dP\left(j\omega \right)}{d\omega }$) of the current patient’s model are firstly computed as functions of the real (*Re*) and imaginary (*Im*) parts of the denominator in the mathematical model from (5):

(15) $$\begin{array}{c} Re\left(j\omega \right)=a_{2}\omega^{\alpha_{2}}\cos\frac{\pi \alpha_{2}}{2}+a_{1}\omega^{\alpha_{1}}\cos\frac{\pi \alpha_{1}}{2}+1\\ Im\left(j\omega \right)=a_{2}\omega^{\alpha_{2}}\sin\frac{\pi \alpha_{2}}{2}+a_{1}\omega^{\alpha_{1}}\sin\frac{\pi \alpha_{1}}{2}\\ M\left(j\omega \right)=\frac{\left|k\right|}{\sqrt{{\left(Re\left(j\omega \right)\right)}^{2}+{\left(Im\left(j\omega \right)\right)}^{2}}} \end{array}$$

(16) $$P\left(j\omega \right)=-\tan^{-1}\frac{Im\left(j\omega \right)}{Re\left(j\omega \right)}-\omega \tau_{m}$$

(17) $$\frac{dP\left(j\omega \right)}{d\omega }=-\left(\frac{\left(a_{2}{\alpha_{2}\omega }^{\alpha_{2}-1}\sin\frac{\pi \alpha_{2}}{2}+a_{1}\alpha_{1}\omega^{\alpha_{1}-1}\sin\frac{\pi \alpha_{1}}{2}\right)Re\left(j\omega \right)}{{\left(Im\left(j\omega \right)\right)}^{2}+{\left(Re\left(j\omega \right)\right)}^{2}}-\frac{Im\left(j\omega \right)\left(a_{2}\alpha_{2}\omega^{\alpha_{2}-1}\cos\frac{\pi \alpha_{2}}{2}+a_{1}\alpha_{1}\omega^{\alpha_{1}-1}\cos\frac{\pi \alpha_{1}}{2}\right)}{{\left(Im\left(j\omega \right)\right)}^{2}+{\left(Re\left(j\omega \right)\right)}^{2}}\right)-\tau_{m}$$

A new structure for the FOPID controller is proposed:

(18) $${\text{C}}_{\text{FOPID}}\left(\text{s}\right)=k_{p}\left(\text{1 +}k_{i}{\text{s}}^{\text{-[GSLL]}}\right)\frac{T_{1}s+1}{T_{2}s+1}$$

where $k_{p}$ and $k_{i}$ are the proportional and integral gains, λ∈(0,2) is the fractional order of integration and *T_1_* and *T_2_* are the time constants of the lead controller. An additional overshoot is associated to the lead controller, with the effective value being directly correlated with the ratio of the two time constants. Given that this controller is specifically intended for drug dosing, it is crucial to minimise the overshoot as any over-dosing can potentially cause significant side-effects. An additional important performance criterion refers to the time-to-target, TT, referring to the amount of time required for the BIS signal to reach the [45], [46], [47], [48], [49], [50], [51], [52], [53], [54], [55] range.

An increase in the ratio *T_1_/T_2_* will decrease the TT, but it will also increase the overshoot. In order to achieve a sufficiently small TT and minimise overshoot, a trade-off must be reached. However, the latter condition is more crucial than the former, leading to the conclusion that *T_1_* and *T_2_* should be restricted to have similar values. Additionally, both *T_1_* and *T_2_* must be directly associated with the gain crossover frequency, *ω_c_*, and they will be computed as a ratio between a constant value and *ω_c_*. Another issue that needs to be addressed is the presence of a non-zero steady-state error in the lead element, which must be eliminated using an integrator. If λ < 1 (indicating a partial integrator), the error will not be rapidly eliminated, resulting in a slower convergence to the steady state of the system [53], [54], [55].

The tuning of the controller in (18) is performed based on a specific set of performance specifications in the frequency domain. These specifications include a desired phase margin to guarantee stability and minimise undershoot of the BIS signal, a target gain crossover frequency to achieve a certain settling time, limitations on the sensitivity and complementary sensitivity to handle any load disturbances or noise, and robustness to variations in gain. The frequency domain representation of the FOPID controller in (18) is obtained as:

(19) $${\text{C}}_{\text{FOPID}}\left(\text{j}\omega \right)=k_{p}\left(\text{1 +}\;k_{i}\omega^{\text{-[GSLL]}}\left(\cos\frac{\lambda \pi }{2}-j\sin\frac{\lambda \pi }{2}\right)\right)\frac{j{\omega T}_{1}+1}{j{\omega T}_{2}+1}$$

with the modulus, phase and derivative of the controller phase computed as:

(20) $$\left|{\text{C}}_{\text{FOPID}}\left(\text{j}\omega \right)\right|=k_{p}\sqrt{{\left(1+k_{i}\omega^{\text{-[GSLL]}}\cos\frac{\lambda \pi }{2}\right)}^{2}+{\left(k_{i}\omega^{\text{-[GSLL]}}\sin\frac{\lambda \pi }{2}\right)}^{2}}∙\sqrt{\frac{{1+\omega }^{2}T_{1}^{2}}{{1+\omega }^{2}T_{2}^{2}}}$$

(21) $${∠\text{C}}_{\text{FOPID}}\left(\text{j}\omega \right)=\tan^{-1}\frac{k_{i}\omega^{\text{-[GSLL]}}\sin\frac{\lambda \pi }{2}}{1+k_{i}\omega^{\text{-[GSLL]}}\cos\frac{\lambda \pi }{2}}+\tan^{-1}{\omega T}_{1}-\tan^{-1}{\omega T}_{2}$$

(22) $$\frac{d∠{\text{C}}_{\text{FOPID}}\left(\text{j}\omega \right)}{d\omega }=-\frac{{\lambda k}_{i}\omega^{\text{-[GSLL]-1}}\sin\frac{\lambda \pi }{2}}{1+2k_{i}\omega^{\text{-[GSLL]}}\cos\frac{\lambda \pi }{2}+k_{i}^{2}\omega^{\text{-2[GSLL]}}}+\frac{T_{1}}{{1+\omega }^{2}T_{1}^{2}}-\frac{T_{2}}{{1+\omega }^{2}T_{2}^{2}}$$

The Phase margin (PM) is a frequently used performance measure that is linked to the stability of the closed loop system and has a direct effect on the predicted overshoot and undershoot. Usually, a high numerical value is employed to indicate a decreased excess. The mathematical equation that addresses the overshoot requirement is:

(23) $$∠{\text{H}}_{\text{OL}}\left(\text{j}\omega_{c}\right)=-\pi +PM$$

where ${\text{H}}_{\text{OL}}\left(s\right)={\text{C}}_{\text{FOPID}}\left(\text{s}\right)H_{\mathrm{DOH}}\left(s\right)$ is the loop transfer function.

The gain crossover frequency, *ω_c_*, indirectly addresses the settling time requirements. Greater values of *ω_c_* are correlated with shorter settling times. Mathematically, this is expressed using the equation for magnitude:

(24) $$\left|H_{\mathrm{OL}}\text{(j}\omega_{c}\text{)}\right|\;=\;$$

The requirement for the controller to handle potential gain variations resulting from possible patient intra-variability is defined as the robustness condition:

(25) $${\left(\frac{{d∠H}_{OL}\left(j\omega \right)}{d\omega }\right|}_{\omega =\omega_{c}}=0$$

Intra-patient variability refers to the distinct and dynamic variations in a patient's physiological traits over time, which occur due to factors such as changing health conditions, fluctuations in metabolism, or variations in individual responses to various treatment protocols. The purpose of designing robust controllers is to address this issue and maintain the closed loop under/overshoot constant despite such variations.

It is essential that both the control signal and the output demonstrate stable behaviour without oscillation. The PM value is chosen in order to achieve the highest possible undershoot, as outlined in (26):

(26) σ≤5%

As stated earlier, the TT performance indicator should be minimised, typically ranging from 3 to 5 min. This condition contradicts (22) as a smaller TT generally results in a larger overshoot. Thus, the gain crossover frequency was selected in a manner that ensures TT meets the specified requirement:

(27) TT≤4minutes

The BIS signal must stay within a range of 40 to 60 at all times. The evaluation of the closed loop results is conducted based on the prevailing performance measures commonly used in closed loop control of anaesthesia, as outlined in the following section.

Each FOPID controller having the form described in (18), specific to each patient, is tuned using an optimization routine that minimizes the error between the actual BIS and the estimated BIS with constraints as specified in (23)-(27). Based on clinical practice, performance indicators for the tuning of the FOPID controller in (18) are imposed [24]. The smallest (BIS-NADIRs) and largest amplitudes (BIS-NADIRl) of the BIS signal should not be lower than 40 or larger than 60 [56]. Propofol rate should remain within 5–200 mg/kg/h [57].

The Matlab “*fmincon*” optimization routine [58] is used to determine the controller parameters. The interior-point algorithm implemented in the “*fmincon*” Matlab function solves constrained nonlinear optimisation problems by converting them into a sequence of unconstrained problems through the use of barrier functions. Rather than explicitly managing constraints, it incorporates a logarithmic penalty term into the objective function to prevent the occurrence of constraint violations. The algorithm iteratively solves the Karush-Kuhn-Tucker (KKT) system using Newton’s method, updating the variables while ensuring feasibility. The solution approaches the true optimum as a barrier parameter μ is progressively reduced. In order to preserve feasibility, step sizes are meticulously selected. This approach is effective for dealing with large-scale issues, effectively managing both convex and non-convex cases. However, it necessitates the resolution of substantial linear systems during each iteration. This entails the identification of the minimum error, i.e. Internal Absolute Error (IAE), while taking into account the specified equations (23)-(27) as the algorithmic conditions. The previously mentioned performance indicators are converted into precise frequency domain performance criteria. The numeric values of these criteria are chosen taking into consideration previous research findings, according to how the patient responded to the nominal controller in [24].

## Results and discussion

A key aspect of the proposed approach refers to the runtime of the algorithms performing the online estimation of the patient model, followed by the online tuning of an individualised controller. The proposed online procedure including modelling, time delay estimation and controller tuning is feasible because the modelling algorithm has a maximum runtime of 25 s, the estimation of the time delay takes a maximum of 2 s, and the optimisation algorithm for tuning the controller has a maximum runtime of 50 s. Modelling can only be performed after the BIS signal has dropped from 100 and a BIS curve is already present. The maximum time allocated for computing the controller parameters is 77 s, which is significantly shorter than the desirable time for hypnosis, which is around 5 min [18]. The proposed controller preset robustness to gain variations so the time delay estimation will ensure that the controller is tuned for the correct delay time and consequently the adaptivity to delay variations is also ensured.

For each patient, the performance specifications referring to phase margin and gain crossover frequency are used to tune the personalised FOPID by solving the system of equations in (23)-(27). For example, for patient 21, the gain crossover frequency has been selected to be *ω_c_* = 0.007 rad/s, while the phase margin is imposed as *PM* = 50°. Similar values are used for the other patients in the database. These are selected similarly to previous research [24], [25], [59]. Using the fractional order model (5) for patient 21 and solving the system of equations (23)-(27) using the “*fmincon*” optimization algorithm, the following parameters for the individualised FOPID controller are obtained: *k_p_* = 0.5093, *k_i_* = 0.0045, *λ* = 0.925, *T_1_* = 142.8571, *T_2_* = 114.2857. For the rest of the patients, the estimation of the FOPID parameters is performed by using the corresponding fractional order patient model and solving the resulting system of equations (23)-(27).

Table 3 provides a comprehensive overview of the results obtained from the closed-loop simulation. This table presents detailed performance metrics and outcomes related to the implementation of the FOPIDs control algorithm in the hypnosis phase. The data presented in this table illustrates the algorithm's efficacy, highlighting substantial enhancements in control accuracy and stability.

**Table 3:** Performance measures for the developed FOPIDs controllers.

Patient no	TT (s)	BIS-NADIRl	BIS-NADIRs
3	144	48.9	50.1
9	218	40.2	53.8
10	172	47.3	51.4
14	152	48.1	50.7
21	225	48.3	51.1
25	315	42.7	59.9
33	179	45.2	51.8
35	195	48.2	53.0
36	386	42.5	55.9
38	126	45.6	52.7
39	340	47.1	52.8
41	181	48.1	54.6
48	216	47.5	51.8
54	120	45.9	51.4
56	298	44.8	55.6
59	192	45.4	51.3
63	346	45.4	56.7
64	169	46.3	50.1
66	154	43.6	55.5
70	194	48.0	50.1

**Fig. 3:**
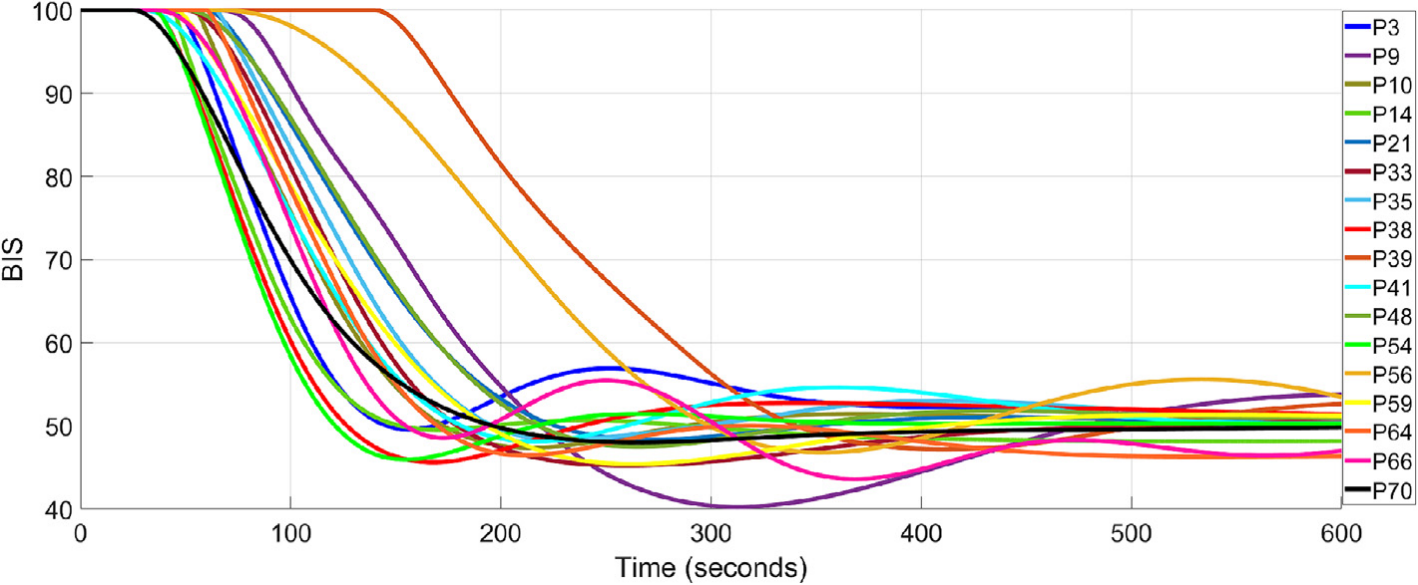
Closed loop simulation results for 17 patients using the online tuned personalised FOPID controllers.

**Fig. 4:**
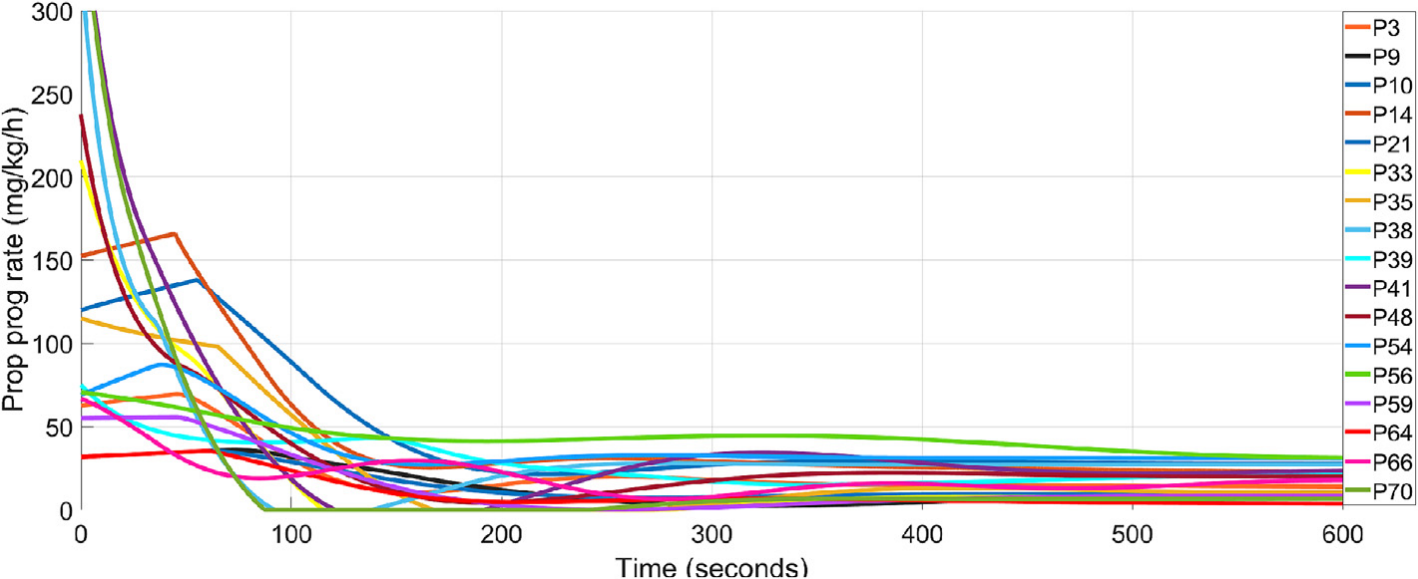
Corresponding control signal as Propofol progression rate for the 17 patients.

Comparing the results in Table 3 with those in [24], where a population-based controller is used, it is evident that online controllers exhibit superior performance. For 17 out of 20 patients, the results indicate a closed loop performance that meets the clinical requirements in (26) and (27). This represents an 85 % success rate, highlighting the suitability of the proposed control strategy, compared to [24] where the success rate was significantly smaller, 36.36 %. The closed loop simulation results for these 17 patients are indicated in Fig. 3, Fig. 4, for the BIS signal and Propofol rate, respectively.

As can be observed, only 2 patients of the 17 have a TT close to 300 s due to their very large dead time constant. As for the rest of the controllers, they exhibit good TT performance.

**Fig. 5:**
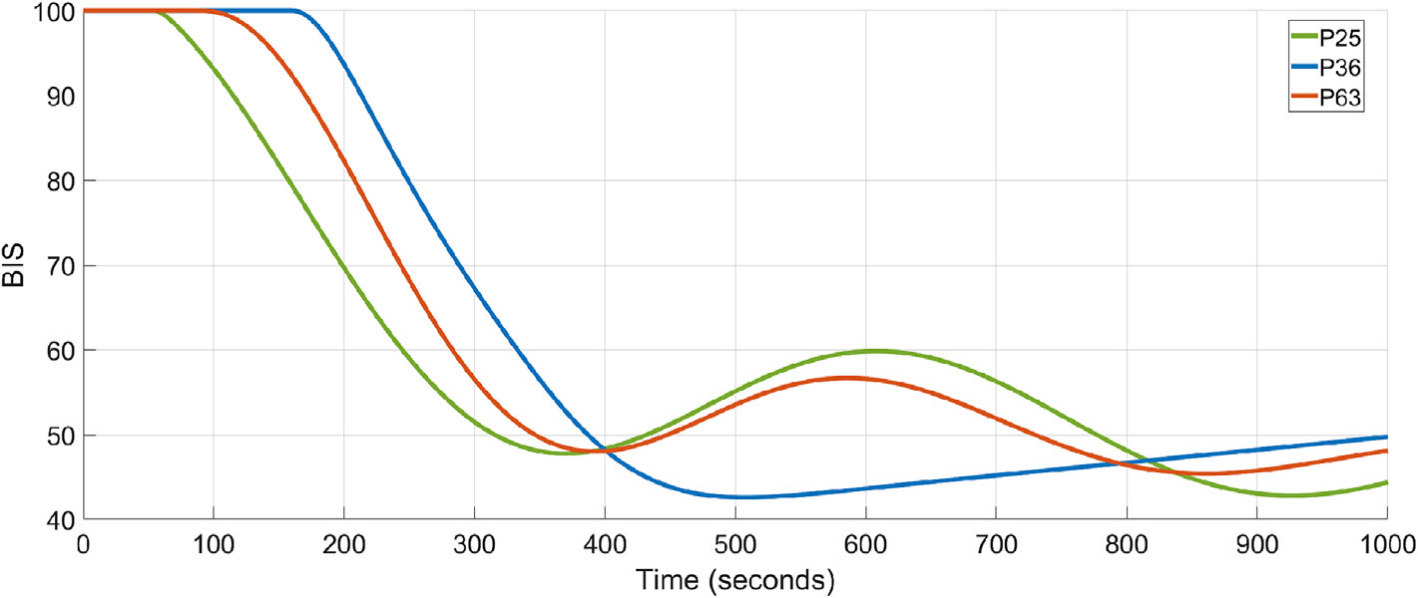
Closed loop BIS response of patients 25, 36 and 63.

In the case of patients 63, 36, and 25, the TT ranges from 320 to 380 s, indicating a relatively sluggish response. This is due to the large time delays observed in these patients, as indicated in Table 2. However, the BIS signal remains within the safe range of 40 to 60, meaning that the personalised controller is able to meet safety standards. Fig. 5 shows a detailed look at the closed loop simulation results for patients 63, 36 and 25.

**Fig. 6:**
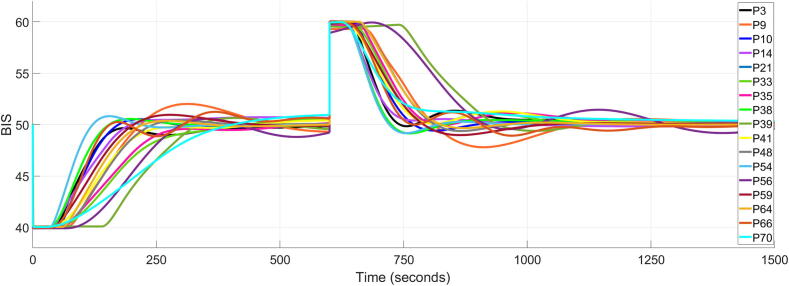
Disturbance rejection simulation results for 17 patients.

The personalised controllers are tuned during the induction phase. The surgical procedure, acting as an output disturbance, begins after the induction phase has ended and corresponds to the maintenance phase of anaesthesia. In what follows, the performance of the personalised FOPID controllers is tested in terms of the rejection capability. A simple disturbance signal is used, similarly to [11] and consists of two step signals of amplitude 10 which occur consecutively with a 10 min delay between them. The first step is a negative one, followed by a positive one. The evaluated performance is indicated by the TT metric, which in this case denotes the amount of time required for the BIS signal to return in the range of 49 to 51, following a disturbance. A tolerance of ± 2 % was considered appropriate since throughout the entire work, the BIS signal was required to remain withing a range of values rather than a strict setpoint. According to the literature [8], [56], accepted values for TT are between 3 and 5 min (180–300 s). Consequently, any value for TT greater than 300 s will be considered unacceptable and oppositely, any value less than 180 s represents a good performance. The simulations results are presented in Fig. 6, for 17 patients, while Table 4 presents the TT values for each patient.

**Table 4:** Disturbance rejection performances.

Patient no	TT for negativestep disturbance(seconds)	TT for positivestep disturbance(seconds)
3	150	120
9	198	192
10	149	147
14	168	131
21	189	190
33	288	167
35	252	174
38	132	110
39	304	307
41	186	161
48	209	187
54	107	110
56	270	283
59	178	166
64	190	190
66	136	140
70	295	180

The simulations in Fig. 6 and the corresponding quantitative performance in Table 4 demonstrate that adequate closed-loop performances were obtained in 16 of the 17 patients. Out of the initial 20 patients, 3 were excluded from this study. As Table 3 and Fig. 5 indicate, the controllers designed for patients 25, 36 and 63 deliver a sluggish response, failing to satisfy the clinical standards. Their disturbance rejection capabilities would be therefore unsatisfactory and therefore these patients were not included in this part of the research. Additionally, the controller for patient 39 failed to meet the required standards, but this is due to the large time delay in the model of 145 s. One could contend that the model is flawed or that the operational measurements were inaccurate, given that this model exhibits nearly 100 s of additional time delay compared to the mean value across all models. The positive outcome is that 6 controllers demonstrated excellent disturbance rejection capabilities. Furthermore, the results indicate that it is easier for a patient to recover from a positive BIS disturbance. The awakening of a patient, corresponding to an increase in the BIS signal, can be mitigated with more ease than the prevention of falling into dangerously deep level of hypnosis. This finding is in accordance with the literature [61. From a control engineering point of view, the control signal (Propofol rate) is always positive and thus an increase in the Propofol rate would diminish the BIS signal and reject the positive disturbance. A negative control signal implies extracting the anaesthetic substance out of the blood and it is impossible in practice, thus making the rejection of a negative disturbance (decrease of BIS signal) more difficult to handle.

The results obtained in this paper are compared to those reported in literature [24], [25], [59], where FOPID controllers are also proposed as optimal control solutions in anaesthesia. The observed overshoot values in both this research and the ones found in literature, exhibit a high degree of similarity, with a deviation of less than 5 %, particularly falling within the range of 3 %–4 %. However, the FOPIDs in [24], [25] and [59] are tuned based on a nominal patient model. The resulting controllers are then used for all other patients in the database. The benefit of using a personalised online tuned FOPID, as reported in this paper, is evident when comparing the TT values during the maintenance phase.

Based on Table 4, the mean value for TT is approximately 187 s. In [24], a FOPID controller is designed for the nominal patient model 21, similarly to this paper. The resulting FOPID is used for 9 different other patients. But the research does not report the TT values during the maintenance phase. In the induction phase, the mean value of TT in [24] is 451 s, significantly higher than the mean TT value of 216 s obtained in this research during the induction phase and reported in Table 3. The mean TT value obtained in [59] is 135 s, whereas in [25] the mean TT value is 128 s, shorter than the value reported in this research. No TT values are reported during the maintenance phase in [25] to compare them with the results obtained with the individualised controllers developed in this paper. The surgical stimulus used in [59] is different than the one in this paper and therefore the comparison is not fair. The better results obtained in [25] and [59] during the induction phase can be explained as follows. The presence of inter-patient variability poses a notable challenge, particularly in the context of tailoring treatments and therapies to individual patients. This is insufficiently tackled in [25] (and also [59]), where a constant and very short time delay is used for each patient, of 19 s. In contrast, in this paper, the patient inter-variability is directly addressed, patients exhibiting time delays ranging from 25 s to 160 s. The constant time delay in [25] and [59] is an important limitation and simplification of the patient variability that render the mean TT value as unrealistic. It additionally allows for a faster TT and an easier design of the controller that does not have to struggle with large time delays, as reported in this research.

Although offline FOPID controllers, such as those in [24], [25] and [59] are effective in many cases, they often struggle to maintain optimal performance when dealing with dynamic physiological responses that are specific to each patient. This is the reason why the online approach provided in this paper proves to be much more suitable. The online adaptation of controllers is necessary to account for inter-patient variability and ensure their suitability for individual patients.

The closed loop maintenance results in Table 4 are compared to an individualised PID control strategy proposed in [12]. The TT values obtained in [12] are approximately 120 s, shorter than the ones reported in Table 4. However, this is due to two aspects: first, the individualised patient model used in [12] has no time delay and second, the approach in [12] is based on a ratio control scheme, where two drugs are considered. These aspects contribute to a faster TT. However, in the case of a positive disturbance, the individualised PIDs in [12] allow the BIS signal to drop to amplitudes as low as 42. In the case of the proposed individualised FOPID controllers, the minimum value of the BIS signal decreases only up to 47, as indicated in Fig. 6. Additionally, the maximum amplitude of the BIS signal following a negative disturbance is 52, similarly to the results in [12]. Thus, the proposed individualised FOPID controllers manage to maintain the BIS signal within a closer range of the reference BIS signal of 50, compared to [12], with the drawback of a larger TT value.

The proposed control approach presents certain challenges that require careful consideration, particularly due to the stringent requirements of its practical application. Given its direct implications for patient health and safety, thorough examination and validation are essential to ensure its reliability. While the individual algorithms used for both modelling and control exhibit numerical stability, the broader algorithmic framework currently lacks formal mathematical guarantees. As a result, establishing a robust validation mechanism would be beneficial in addressing potential instances where the proposed algorithms may not generate optimal controllers. Such a mechanism could enhance reliability by either refining the controller selection process or providing timely notifications to medical personnel when adjustments are needed. The offline evaluation of control performance conducted in this study provides valuable insights, and future efforts should focus on developing a stable online verification system to further support real-world applications. While this method offers improvements over comparable alternatives in the existing literature, there is still room for improvement, particularly in incorporating fundamental safety measures that are crucial for real-world deployment. This study was conducted on a limited offline set of data. A larger database would be considerably beneficial for this research. The significance and reliability of the results and conclusions are greatly enhanced when the database utilized contains a substantial number of samples.

## Conclusion

The study highlights the use of a FOPID controller to effectively regulate the Depth of Hypnosis during the induction and maintenance process. This novel approach deviates from conventional PK-PD models and employs fractional order models for the purpose of controller design. These fractional order models are estimated during the induction phase. An online estimation of the time delay is also implemented. As such, the study highlights the substantial fluctuations in the fractional order models, caused by patient variability, which can a significant impact on the closed loop performance if not properly accounted for.

The estimated fractional order time delay model is further used to design individualised FOPID controllers. The parameters of these FOPID controllers are estimated online using an optimization routine. The findings demonstrate the effectiveness of the personalised FOPID controllers, with mean values of 4 % for the undershoot and a commendable mean value of TT of 192 s. Furthermore, the fact that the results were acceptable for 3 out of 20 patients and great for 17 out of 20 emphasises the efficiency of the suggested control approach.

Further research includes the following. The developed technique should be validated on a patient simulator [48], including interactions from other drugs and hemodynamic variables. The research in this paper could potentially represent the starting point for a multivariable approach similarly to [45]. Another issue refers to the implementation of different online model estimation algorithms as in [46], [47] and comparing the results with those obtained in this paper. Additionally, a faster online estimation of the model would be beneficial, using fewer samples from the induction phase. The online model estimation algorithm used in the paper could be combined with a model predictive control scheme [60] a control algorithm that resembles the actions of the anaesthesiologist.

## CRediT authorship contribution statement

**Marcian Mihai:** Software, Investigation, Validation, Methodology. **Isabela Birs:** Software, Methodology. **Erwin Hegedus:** Software, Methodology. **Amani Ynineb:** Software, Methodology. **Dana Copot:** Writing – review & editing. **Robain De Keyser:** Software. **Clara M. Ionescu:** Methodology, Writing – review & editing. **Samir Ladaci:** Methodology. **Cristina I. Muresan:** Conceptualization, Supervision, Software, Writing – review & editing. **Martine Neckebroek:** Methodology, Writing – review & editing, Investigation.

## Declaration of competing interest

The authors declare that they have no known competing financial interests or personal relationships that could have appeared to influence the work reported in this paper.
